# Factors Associated with the Dimensions of Compassion Fatigue in Nursing: The Role of Empathy and the Work Environment

**DOI:** 10.3390/healthcare14193132

**Published:** 2026-09-22

**Authors:** Roberto Zegarra-Chapoñan, Jhon Alex Zeladita-Huaman, Rosa Castro-Murillo, Flor De Jeanette Blas-Bergara, Eduardo Franco-Chalco, Elito Mendoza-Quijano, Henry Castillo-Parra, Gabriela Samillan-Yncio, Laryn Smith

**Affiliations:** 1 Faculty of Health Science, Universidad María Auxiliadora, Lima 15408, Peru; rob.zegarra@gmail.com; 2 Academic Department of Nursing, Faculty of Medicine, Universidad Nacional Mayor de San Marcos, Lima 15001, Peru; gsamillani@unmsm.edu.pe; 3 Mental Health and Psychiatry Service, Hospital Hermilio Valdizan, Lima 15011, Peru; rosacastromurillo@gmail.com; 4 Epidemiology and Environmental Health Unit, Hospital San Juan de Lurigancho, Lima 15538, Peru; flordejeanetteblas@gmail.com; 5 Academic Department of Psychology, Pontificia Universidad Católica del Perú, Lima 15008, Peru; 6 Academic Department of Public Health, Universidad Nacional Toribio Rodríguez de Mendoza de Amazonas, Chachapoyas 01001, Peru; elito.mendoza@untrm.edu.pe; 7 Faculty of Psychology, Universidad de San Buenaventura, Medellín 050021, Colombia; gerencia@neuromind.net; 8 Nursing Doctoral Program, Faculty of Medicine, Universidad Nacional Mayor de San Marcos, Lima 15001, Peru; laryn12@gmail.com

**Keywords:** nursing, compassion fatigue, empathy, working conditions, personal satisfaction

## Abstract

**Background/Objectives:** Compassion fatigue can affect nursing professionals exposed to demanding clinical environments and may be influenced by individual and work-related factors. The objective of this study was to analyze the factors associated with the compassion and fatigue/exhaustion dimensions of compassion fatigue (CF) among nursing professionals in Peru. **Methods:** This was an observational, analytical, and cross-sectional study in 374 nursing professionals from Peru, with data on the study variables collected at a single point in time through a virtual survey. Primary analyses used the two original EFat-Com subscales and complete-case multiple linear regression without automated selection (*n* = 339), with HC3 robust 95% confidence intervals (CIs). Sensitivity analyses examined age, resilience, continuous life satisfaction, workplace grouping, and an exploratory 11-item version; stepwise models underwent bootstrap validation. **Results:** The sample was predominantly female (88.2%) and had a mean age of 39.77 years (SD = 9.11). Empathy was associated with compassion (b = 0.312; 95% CI 0.237 to 0.387); adjusted R^2^ was 0.216. Experience exceeding ten years (b = −0.201; 95% CI −0.396 to −0.006) and neutral job satisfaction (b = 0.226; 95% CI 0.057 to 0.395) were associated with fatigue/exhaustion scores; adjusted R^2^ was 0.080. Several associations varied across sensitivity analyses, whereas the empathy–compassion association persisted. **Conclusions:** The dimensions showed different association patterns, but the fatigue/exhaustion model had limited explanatory capacity, suggesting that other relevant factors not assessed in this study may also contribute to this dimension. Measurement uncertainty, missing data, and model instability warrant cautious interpretation.

## 1. Introduction

In nursing practice, continuous contact with suffering, trauma, and emotional demands of care can increase susceptibility to compassion fatigue (CF), especially in highly complex clinical settings [1,2]. This phenomenon is a relevant occupational health problem due to its association with alterations in physical and psychological well-being, lower work performance, and intention to leave the job [1,2]. A systematic review with meta-analysis of 196 studies reported moderate levels of burnout and secondary traumatic stress in nursing professionals, with higher scores in intensive care units and emergency departments, although with high heterogeneity among the included studies [1].

CF has been described as a state of physical and emotional exhaustion that may be accompanied by psychological and spiritual disturbances and with reduced ability to empathize with and tolerate the suffering of others [3,4,5]. This construct was first used in the nursing literature by Joinson [6] in 1992, who used it to describe a form of emotional exhaustion observed in nurses repeatedly exposed to their patients’ suffering. Subsequently, Figley [3] developed and systematized this concept as one of the “costs of caregiving”, linked to direct and prolonged exposure to the suffering of other people and to the empathic response of the professional. While burnout is usually related to chronic work demands and unfavorable organizational conditions, CF is more specifically linked to empathic exposure to the patient’s suffering and trauma. Although some authors describe a relatively rapid onset of its manifestations, it can also develop cumulatively with repeated exposures [3,7,8].

In the literature, there is no consensus on the definition and dimensionality of CF. One of the instruments most widely used by the scientific community is the Professional Quality of Life Scale (ProQOL), which distinguishes a positive component (compassion satisfaction) and two negative components (burnout and secondary traumatic stress) [9,10]. In the Peruvian context, the Compassion Fatigue Scale (EFat-Com) was developed, consisting of two factors: compassion and fatigue/exhaustion [11]. In this study, the original EFat-Com dimension labels are retained; therefore, the compassion dimension is interpreted as a positive component related to empathic disposition and the favorable experience of care, but it is not assumed to be an exact equivalent of compassion satisfaction assessed using the ProQOL. The fatigue/exhaustion dimension is interpreted as a negative emotional response to the suffering of others, characterized by emotional exhaustion and frustration associated with prolonged exposure to compassion-related stress. These two dimensions may show distinct patterns of association, while their relationship within a broader overarching construct remains an empirical question, which supports examining their associated factors separately [12].

Compassion involves recognizing and responding to another person’s suffering with the intention of alleviating it [13]. In contrast, the fatigue/exhaustion dimension of the EFat-Com reflects a negative emotional response associated with prolonged exposure to suffering in caregiving contexts [11]. Compassion satisfaction refers to the positive experience and satisfaction derived from helping others [10], whereas burnout is primarily associated with chronic occupational stress [14]. Secondary traumatic stress refers to stress responses arising from indirect exposure to another person’s traumatic experiences [14]. Although these constructs may coexist and be related, they are conceptually distinct and were therefore differentiated when interpreting the findings of this study.

Among personal factors, empathy occupies an important place due to its dual function: it constitutes an essential clinical competence for patient-centered care and, at the same time, some of its dimensions may be associated with greater emotional vulnerability [15,16]. In the stress and CF model, empathy facilitates the connection with the patient’s suffering and can favor vicarious exposure to trauma; however, this relationship varies according to the empathic component considered [17,18]. Cognitive empathy and perspective-taking have been associated with lower CF and secondary traumatic stress, while personal distress and certain forms of affective involvement have been related to higher levels of fatigue, secondary traumatic stress, and burnout [16,18]. Consequently, the evidence does not support a uniform effect of empathy; its relationship with CF seems to depend on the form of empathic processing and contextual resources such as social support [17].

Several authors have examined the relationship between sociodemographic and occupational characteristics such as sex, age, professional experience, clinical unit, and job satisfaction with the dimensions of CF; however, the magnitude and direction of these associations are not uniform [10,19,20]. The differences observed between geographical regions, clinical services, and professional groups can be related to the heterogeneity of the work contexts, measurement instruments, sampling strategies, and analytical methods used [1,19]. In contrast, evidence more consistently shows that the practice environment, psychological demands, leadership, job satisfaction, and resources such as social support, self-efficacy, self-compassion, and emotional intelligence are associated with burnout and secondary traumatic stress [12,21,22,23,24]. Taken together, these findings support a multifactorial and context-dependent understanding, although predominantly cross-sectional designs preclude establishing causal relationships or definitive interactions.

Beyond occupational factors specific to caregiving, psychological well-being may also be understood within a broader multidimensional framework involving behavioral, social, biological, and environmental determinants. Recent evidence from other populations has highlighted the relevance of behavioral and social factors, including sleep, physical activity, alcohol consumption, and patterns of social interaction, to mental health outcomes [25]. Likewise, psychiatric and physical health may be interconnected through biological, behavioral, and environmental pathways [26]. Although these studies address populations and outcomes different from compassion fatigue in nursing, they reinforce the broader relevance of considering multiple individual and contextual determinants when examining psychological well-being.

A broader understanding of compassion fatigue may also consider personal resources and work-related psychosocial factors. From a salutogenic perspective, sense of coherence (SOC), comprising comprehensibility, manageability, and meaningfulness, has been associated with lower work-related stress and adverse psychological outcomes and with greater well-being among care professionals [27]. Perceived social support has also been associated with lower compassion fatigue and burnout and with greater compassion satisfaction among nurses [28]. Emotional intelligence represents another relevant personal resource, with higher levels associated with greater compassion satisfaction and lower compassion fatigue among nurses [12]. Similarly, resilience has been associated with prosocial behavior among professional caregivers, highlighting the potential relevance of personal psychological resources in demanding care contexts [29]. Collectively, these findings support considering both personal and contextual resources when examining occupational outcomes among care professionals and are consistent with the Conservation of Resources (COR) perspective adopted in the present study [30].

Although multivariate models have been developed on CF, most have focused on specific domains such as personal variables, job characteristics, or organizational factors, predominantly using cross-sectional designs and particular clinical populations [1,12,21,22]. Consequently, there is still limited evidence that simultaneously examines, within the same population and through a common analytical framework, the associations of empathy, sociodemographic and occupational characteristics, and the practice environment with the two dimensions of EFat-Com. In this context, it is methodologically pertinent to estimate independent models for the compassion and fatigue/exhaustion dimensions to determine whether they exhibit different patterns of association.

Considering these gaps, COR theory provides a relevant interpretive framework, proposing that well-being and attrition can be related to the availability, threat, or loss of personal and work resources [30]. From this perspective, it is plausible that the factors associated with the compassion dimension are not identical to those related to the fatigue/exhaustion dimension. In this context, the present study aimed to analyze the factors associated with the compassion and fatigue/exhaustion dimensions of CF among nursing professionals in Peru.

## 2. Materials and Methods

### 2.1. Study Design

An observational, analytical, cross-sectional study was conducted from 8 July 2025 to 29 September 2025. The COR Theory was used as an interpretative framework to examine the associations of personal and work resources with study outcomes; however, the study did not directly measure processes of loss, gain, or investment of resources [30].

### 2.2. Population, Sample, and Sampling

The study population comprised nursing professionals working in healthcare facilities in Lima. Nurses who worked in hospitals, clinics, and health facilities with hospitalization services in Peru, and who had access to electronic devices with an internet connection to complete the virtual questionnaire, were eligible. Nurses who resided in another country, those who performed exclusively administrative functions, and those who reported a previous diagnosis of a mental health problem, such as depression, anxiety, or other related disorders, were excluded. The recorded sample also included respondents reporting no current institutional employment; their exclusion was examined as a sensitivity analysis because this response does not establish a current clinical role.

The sample size was estimated according to the multiple linear regression model with 10 model parameters, assuming a 5% significance level, 80% power, and a medium effect size (f^2^ = 0.15) according to Cohen’s criteria. A minimum of 118 participants was required. After accounting for a potential 20% loss due to incomplete records, the minimum sample size was increased to 142 nursing professionals. Convenience non-probability sampling was used. The data file contained 386 records. One record indicating residence outside Peru and two records excluded during the original response-quality review were removed. Nine additional records lacked sociodemographic responses: three declined consent, and six consented but provided no sociodemographic responses. Thus, 374 participants contributed sociodemographic information. The original sample-size calculation did not specifically evaluate the larger number of indicator coefficients in the revised full models.

### 2.3. Variable and Instruments

Information collection was carried out through a self-administered virtual survey. The questionnaire was implemented on the AllCounted platform, selected for its security and privacy features, including tools to prevent unauthorized responses, restrict access through passwords or codes, and control the sharing of results.

The first section collected sociodemographic variables (age, sex, religious affiliation, and place of residence) and labor variables (type of health facility in which they work and years of experience).

Empathy was assessed using a specific factor of the Communication Skills Scale adapted for Peruvian nurses. This scale, originally developed in Spain, was subjected to a process of cultural adaptation to the Peruvian context, with review and adjustment of items through a focus group. In the validation study, the empathy subscale showed factor loadings greater than 0.62 and adequate internal consistency, with a McDonald’s omega (ω) coefficient of 0.82 [31]. The empathy subscale consists of five items rated on a 6-point Likert-type scale ranging from 1 (almost never) to 6 (very many times). Higher scores indicate higher levels of empathy. In the current sample, the five empathy items were coded from 1 to 6 (*n* = 340); conventional α = 0.896, ordinal α = 0.924, and ω = 0.896. The one-factor model yielded scaled CFI = 0.990, TLI = 0.980, RMSEA = 0.182, and SRMR = 0.025, indicating discordant fit indices despite high reliability.

Psychological resilience was assessed using the abbreviated version of the Connor–Davidson Resilience Scale (CD-RISC-10), recently validated in a sample of Peruvian nurses [32]. The 10 items were coded from 1 (not true at all) to 5 (true nearly all the time), and their mean was used in the sensitivity analyses. In the current sample (*n* = 370), conventional α was 0.823, ordinal α was 0.888, and model-based ω was 0.842. The one-factor WLSMV model yielded scaled CFI = 0.945, TLI = 0.929, RMSEA = 0.126, and SRMR = 0.059; therefore, reliability should not be interpreted as evidence of uniformly satisfactory structural fit.

The nursing work environment was assessed with the Practice Environment Scale of the Nursing Work Index (PES-NWI), composed of 31 items distributed across five subscales: nurse participation in hospital affairs, nursing foundations for quality of care, nursing manager’s skill/leadership/support, staff and resource adequacy, and nurse–doctor collegiate relations. The scale employed a Likert format of four response options, ranging from 1 (strongly disagree) to 4 (strongly agree). The scale showed adequate reliability in the Hispanic population, with α of 0.89, and high internal consistency in Peruvian nurses, with ω of 0.95 [33]. The 31-item mean was used. In the current sample (*n* = 340), conventional α = 0.950 and ordinal α = 0.962. The five-factor CFA had an inadmissible latent correlation of 1.016 between participation and leadership/support, with a non-positive-definite latent covariance matrix; its model-based ω was therefore not interpreted as a valid estimate.

Life satisfaction was assessed using the Satisfaction With Life Scale (SWLS), originally developed by Diener et al. [34] and psychometrically validated in the Peruvian context [35]. The five items used a five-point response format from strongly disagree to strongly agree. The item mean was analyzed continuously, without categorization. In the current sample (*n* = 362), conventional α was 0.838, ordinal α was 0.889, and ω was 0.844; scaled CFI = 0.998, TLI = 0.996, RMSEA = 0.057, and SRMR = 0.014. Job satisfaction was a separate single question about the respondent’s work or principal workplace, with satisfied, neither satisfied nor dissatisfied, and dissatisfied response categories. The categories in Table 1 and the primary regressions refer to this job-satisfaction question, not to the SWLS.

CF was assessed using the EFat-Com, developed in Peruvian nursing professionals [11]. The administered version was composed of 13 items, originally distributed across two dimensions: tiredness or fatigue-exhaustion and compassion. The items are answered using a five-point Likert scale (1 = “Never” to 5 = “Always”). In the original study, favorable evidence of content validity and internal structure was reported, as well as an internal consistency of α = 0.807 for the total score. Because in the present study the dimensions were used as separate dependent variables, their internal structure and reliability in the analyzed sample were examined. Primary analyses preserved all 13 items, while the modified 11-item scoring was examined only as an exploratory sensitivity analysis.

### 2.4. Procedures

The virtual questionnaire was disseminated through institutional social networks of hospitals and healthcare facilities, as well as through universities with postgraduate programs enrolling nursing professionals. Data collection was conducted using the AllCounted platform.

### 2.5. Analysis

The original 13-item EFat-Com was retained for the primary analyses: compassion was the mean of items 7–13 and fatigue/exhaustion the mean of items 1–6. No overall CF score was computed. CFA used WLSMV for ordinal indicators and complete cases. We examined one- and two-factor models with 13 items, one- and two-factor models with 11 items, and the previously explored 11-item model allowing residual covariances for item pairs 1–2, 11–13, 11–2, and 10–4. Removing items 6 and 12 and freeing these covariances were exploratory, data-informed modifications, not a priori specifications. Accordingly, the modified scores were used only in sensitivity analyses. We report scaled χ^2^, CFI, TLI, RMSEA with 90% CIs, and SRMR, distinguishing conventional and robust alternatives. Models with different item sets were not compared using ordinary χ^2^ differences or AIC. Conventional α, ordinal α, and model-based ω were examined; ω was not interpreted when the factor solution was inadmissible.

Descriptive statistics used available observations and Spearman correlations used available pairs. Scale means required complete item responses; no imputation was performed. Available age, resilience, and SWLS values were compared between complete and incomplete analytic cases using Wilcoxon rank-sum tests. The two primary linear regressions included the entire original candidate set: sex, professional experience, religion, establishment type, job satisfaction, empathy, and practice environment. Categorical covariates were represented by indicators, with male sex, less than five years of experience, Catholic religion, hospital employment, and satisfied job status as reference categories. The full models were adopted during revision to avoid relying solely on automated selection; they were not prospectively preregistered. Both used the same 339 complete cases, and all included coefficients are reported in Section 3.

Inference used HC3 heteroscedasticity-robust standard errors, two-sided t tests, and 95% CIs. Standardized coefficients were calculated as b × SD(covariate)/SD(outcome), including the observed SD of binary indicators. We assessed heteroscedasticity using the Breusch–Pagan test, collinearity using VIF/GVIF, and influence using Cook’s distance, without treating D < 1 as proof of no influence. Sensitivity models added age, resilience and continuous SWLS; grouped establishments as hospital, health center, or other; omitted job satisfaction or experience in separate specifications; excluded respondents reporting no institutional employment; and repeated analyses with 11-item scores. These analyses were defined during revision, not before data collection. Age and experience were retained together to examine their distinct adjusted associations. All revised analyses used R 4.5.3, lavaan 0.6–21, and semTools 0.5–8.

For comparability, the original establishment categories were retained in the primary models, but estimates from very small cells were treated as exploratory. Workplace grouping in sensitivity models reduced the influence of sparse indicator categories; the “other” group combined clinics, polyclinics, independent practice and non-institutional employment and was not interpreted as a homogeneous setting. We also reproduced the earlier stepwise procedure: bidirectional selection started from an intercept-only model within the full candidate set and minimized AIC (k = 2), adding or removing whole terms. Its instability was examined with 500 participant-level bootstrap samples per outcome and scoring version (seed 20260906), repeating selection in each sample. Optimism was the mean difference between bootstrap-training R^2^ and R^2^ obtained by applying that model to the original complete-case sample; this was subtracted from the apparent R^2^. Only successful fits and predictions were summarized. This validation concerned regression selection with fixed scores, not the exploratory CFA search.

### 2.6. Ethical Aspects

The study was approved by the Ethics and Research Committee of Universidad María Auxiliadora (Acta No. 02-2025). Participation was voluntary, and access to the questionnaire was preceded by an electronic informed consent process. Participant confidentiality and data privacy were maintained throughout the study.

## 3. Results

Participants ranged in age from 21 to 69 years (M = 39.77; SD = 9.11), and 88.24% were female. Regarding professional experience, 47.33% reported more than 10 years of experience, whereas 22.46% had less than five years of experience. As for religious affiliation, a predominance of Catholic identity was observed, followed by Evangelical Christianity. Similarly, 74.06% of the population reported living in the city of Lima. Regarding workplace setting, most participants worked in hospitals (62.88%). Finally, when analyzing the perception of job satisfaction, more than two-thirds of the sample expressed satisfaction, with minimal levels of job dissatisfaction (Table 1).

Of the 374 participants, 13 (3.48%) lacked both establishment and job-satisfaction responses. Resilience was available for 370, SWLS for 362, complete practice-environment and empathy scores for 340 each, and EFat-Com scores for 339 (35 missing; 9.36%). Age was available for 373. All primary and expanded regressions used the same 339 complete cases. The increasing loss across questionnaire blocks was compatible with incomplete participation or survey routing, but individual reasons were not recorded. All 11 respondents selecting “other” establishment lacked complete EFat-Com data; 19 of 21 reporting no institutional employment entered the regressions. Comparisons of available age, resilience, and SWLS between complete and incomplete analytic cases yielded *p* = 0.462, 0.896, and 0.160, respectively; these findings do not establish that missingness was completely at random.

CFA of the EFat-Com included 339 participants. The original two-factor model showed inadequate absolute fit: scaled χ^2^(64) = 526.307, *p* < 0.001, CFI = 0.914, TLI = 0.895, RMSEA = 0.146 (90% CI 0.135–0.158), and SRMR = 0.115. Its conventional indices were χ^2^(64) = 549.245, CFI = 0.954, and RMSEA = 0.150. CFI measures improvement over a baseline model, whereas RMSEA reflects discrepancy relative to degrees of freedom; their divergence does not establish acceptable fit. The 13-item one-factor solution fit worse descriptively (scaled χ^2^(65) = 2075.075, CFI = 0.624, RMSEA = 0.302, SRMR = 0.214). For the original compassion and fatigue/exhaustion subscales, conventional α was 0.881 and 0.801, ordinal α was 0.919 and 0.834, and model-based ω was 0.894 and 0.823, respectively. These reliability estimates are conditional on an imperfect measurement model.

Among 11-item models, the one-factor solution yielded scaled χ^2^(44) = 1698.340, CFI = 0.581, RMSEA = 0.334, and SRMR = 0.190; the two-factor model without residual covariances yielded scaled χ^2^(43) = 310.902, CFI = 0.932, RMSEA = 0.136, and SRMR = 0.097. The exploratory modified model improved fit (scaled χ^2^(39) = 153.413, *p* < 0.001, CFI = 0.971, TLI = 0.959, RMSEA = 0.093 [90% CI 0.078–0.109], SRMR = 0.072), but residual misfit remained. Its conventional CFI/RMSEA was 0.986/0.086 and robust alternatives were 0.890/0.129. The latent correlation changed from −0.138 in the original model to −0.015 after item removal and approximately −0.004 after residual covariances were added. For the retained compassion/fatigue subscales, ordinal α was 0.905/0.798 and ω was 0.850/0.706. The original 13-item scoring was retained as primary because the modifications require independent replication.

Table 2 details the descriptive statistics and the correlation matrix for the study variables. The mean nursing practice environment score was 2.70 (SD = 0.57). Because this value was above the theoretical cutoff of 2.50, it suggested a generally favorable perception of the practice environment. On the other hand, the mean empathy score was 4.76 (SD = 0.82), with scores ranging from 2.40 to 6.00. The mean scores for compassion and fatigue/exhaustion dimensions were 4.13 (SD = 0.61) and 2.78 (SD = 0.69), respectively. Descriptive *n* was 340 for environment and empathy and 339 for both EFat-Com dimensions. Pairwise *n* was 340 for environment–empathy and 339 for the other correlations in Table 2.

The analysis of associations using Spearman’s rho coefficient revealed a positive and statistically significant correlation between the practice environment and empathy (rho = 0.23, *p* < 0.001). Likewise, a positive relationship was found between the practice environment and the compassion dimension (rho = 0.12, *p* < 0.05). Additionally, a positive association of moderate magnitude was observed between empathy and the compassion dimension (rho = 0.41, *p* < 0.001). None of the fatigue/exhaustion correlations shown in Table 2 were statistically significant. Resilience had M = 4.03 (SD = 0.54; *n* = 370) and correlated positively with compassion (ρ = 0.326) and negatively with fatigue/exhaustion (ρ = −0.274). Continuous SWLS had M = 3.89 (SD = 0.65; *n* = 362), with corresponding correlations of 0.331 and −0.275. These four correlations used *n* = 339 and all had *p* < 0.001.

The full primary compassion model included 339 cases (Table 3), with R^2^ = 0.253 and adjusted R^2^ = 0.216. Evidence of heteroscedasticity was present (Breusch–Pagan *p* < 0.001), supporting the use of HC3 inference. The largest Cook’s distance was 0.118; this does not exclude influence from individual observations.

Empathy was positively associated with compassion (b = 0.312; β = 0.419; 95% CI 0.237–0.387; *p* < 0.001). Experience exceeding ten years was also associated with higher scores relative to less than five years (b = 0.176; 95% CI 0.010–0.341; *p* = 0.038). Female sex had a positive estimate, but its robust CI included zero (b = 0.202; 95% CI −0.016 to 0.421; *p* = 0.070). The lower estimate for polyclinic employment (b = −0.523; 95% CI −0.977 to −0.070; *p* = 0.024) was based on only five participants and should not support a firm setting-specific conclusion. The remaining coefficients are reported in Table 3.

The full primary fatigue/exhaustion model also included 339 cases (Table 4), with R^2^ = 0.123 and adjusted R^2^ = 0.080. Thus, the model accounted for a relatively small proportion of the variability in fatigue/exhaustion scores, indicating that a substantial proportion of the variability remained unexplained by the variables included in the model. The Breusch–Pagan test gave *p* = 0.144, and the maximum Cook’s distance was 0.130. HC3 inference was retained consistently across outcomes.

Experience exceeding ten years was associated with lower fatigue/exhaustion scores (b = −0.201; β = −0.147; 95% CI −0.396 to −0.006; *p* = 0.043). Neutral job satisfaction was associated with higher scores relative to being satisfied (b = 0.226; β = 0.148; 95% CI 0.057–0.395; *p* = 0.009). Neither practice environment (b = −0.129; 95% CI −0.289 to 0.030; *p* = 0.112) nor health-center employment (b = 0.216; 95% CI −0.018 to 0.451; *p* = 0.070) met the nominal 0.05 threshold with robust inference. The dissatisfied job-satisfaction category had a wide interval (b = −0.017; 95% CI −0.407 to 0.373); this does not demonstrate equivalence or a non-monotonic effect.

Age and experience were strongly related (Spearman’s ρ = 0.704; *n* = 373; *p* < 0.001), but age had VIF = 1.85 in the expanded models. With age, resilience, continuous SWLS, and grouped workplace categories added (*n* = 339), adjusted R^2^ was 0.233 for compassion and 0.111 for fatigue/exhaustion. Empathy remained associated with compassion (b = 0.277; 95% CI 0.195–0.360). Resilience was associated with higher compassion (b = 0.178; 95% CI 0.048–0.307) and lower fatigue/exhaustion (b = −0.191; 95% CI −0.339 to −0.043). The experience >10 years coefficient for fatigue attenuated to −0.028 (95% CI −0.279 to 0.222); the environment coefficient was −0.083 (95% CI −0.240 to 0.074). SWLS was included as a continuous, separate measure; its CIs included zero for both outcomes. These expanded analyses were exploratory and should not be interpreted as prospective confirmation.

With the 11-item scores, expanded-model adjusted R^2^ was 0.218 for compassion and 0.091 for fatigue/exhaustion. Empathy remained associated with compassion (b = 0.272; 95% CI 0.188–0.357); resilience remained positively associated with compassion (b = 0.189; 95% CI 0.056–0.323) and negatively associated with fatigue/exhaustion (b = −0.179; 95% CI −0.328 to −0.030). Mean scores changed to 4.07 (SD = 0.63) for compassion and 2.92 (SD = 0.70) for fatigue. Excluding the 19 non-institutionally employed respondents from the expanded 13-item models (*n* = 320) gave adjusted R^2^ = 0.227 and 0.099; the empathy–compassion association (b = 0.280; 95% CI 0.194–0.366) and both resilience associations persisted. These sensitivity analyses do not resolve the eligibility or measurement limitations.

The reproduced stepwise models had adjusted R^2^ = 0.213 and 0.083 for the original compassion and fatigue/exhaustion scores. Of 500 bootstrap attempts per outcome, 499 and 495 produced valid fits and predictions. Empathy entered 100% of valid compassion models, compared with 72.9% for sex and 68.5% for experience. For fatigue/exhaustion, job satisfaction entered 87.3%, experience 74.3%, and practice environment 65.7%. Frequencies refer to entire terms, not individual-category significance. Apparent unadjusted R^2^ values of 0.222 and 0.110 fell to optimism-corrected estimates of 0.158 and 0.018. For the 11-item versions, 494 and 497 attempts were valid and corrected R^2^ values were 0.147 and −0.022; a negative corrected value indicates very poor estimated generalization. Failed attempts were omitted, and these estimates are internal validation rather than external replication.

## 4. Discussion

The original EFat-Com dimensions showed different association patterns in full models. Empathy was consistently associated with compassion. Experience exceeding ten years and neutral job satisfaction were associated with fatigue/exhaustion in the primary model, but several associations weakened or varied in sensitivity analyses. Female sex, practice environment, and health-center employment did not have robust CIs excluding zero in the primary models. Thus, conclusions based solely on the earlier stepwise models were not uniformly supported by the revised analyses.

Empathy was consistently associated with compassion, whereas its fatigue/exhaustion coefficient was close to zero with a CI spanning zero. This difference in estimates does not establish different causal effects. However, a study conducted in Turkey during the COVID-19 pandemic reported that empathy is not associated with CF [5]. The literature shows that cognitive empathy and perspective-taking have been associated with less fatigue or secondary traumatic stress, while personal distress and certain forms of affective involvement are related to greater burnout and secondary traumatic stress [16,18]. However, these empathy components were not assessed separately in the present study, which used a unidimensional empathy subscale; therefore, the observed association cannot be attributed to any specific empathic component. A study conducted in China found a positive association between empathy and fatigue partially mediated by social support, confirming the heterogeneity of the results [17], further illustrating the heterogeneity of findings across studies. Accordingly, the present results support an association between the empathy score assessed in this study and the compassion dimension but do not allow conclusions about the specific cognitive or affective mechanisms underlying this association. Although empathy and compassion are conceptually distinguishable constructs, they may share content related to understanding and responding to others’ suffering [36]. This conceptual proximity is particularly relevant because the EFat-Com compassion dimension includes content related to empathic disposition. Therefore, part of the magnitude of the observed association may reflect shared conceptual content between the empathy measure and the compassion dimension rather than an association between fully distinct constructs. Future studies should examine the discriminant validity of these measures.

The estimate for female sex was positive, but its robust CI included zero in the primary compassion model and its statistical significance varied across specifications. Positive estimates for women have also been reported for compassion satisfaction among American nurses [37], although that construct is not identical to EFat-Com compassion. However, the available evidence is inconsistent. A study conducted in Malaysia determined that the initial difference by sex ceased to be significant after incorporating factors from the work environment [38]. Another study conducted in Saudi Arabia reported that gender was not a predictor of compassion satisfaction, but it was a predictor of burnout [20]. Consequently, the evidence suggests that sex-related associations with positive compassion-related outcomes may be contextually dependent and partially explained by work and organizational differences; however, cross-sectional designs and the low representation of men prevent establishing the underlying mechanism.

The two-factor EFat-Com models fit better descriptively than the corresponding one-factor models, but neither the original nor the exploratory modified specification provided unequivocally satisfactory fit. The original subscales were retained for primary scoring to avoid replacing the instrument with an unreplicated, data-derived version. Removing items 6 and 12 and freeing residual covariances were exploratory changes. Item 12 refers to feeling good about helping and may capture self-reward, whereas item 6 explicitly addresses emotional exhaustion and its removal is less readily justified by content alone. Item pairs 1–2 and 11–13 share some thematic content; the cross-factor residual associations 11–2 and 10–4 are harder to explain without additional evidence. These possible interpretations are not evidence of a priori rationale. The near-zero factor correlation depended on respecification and cannot establish conceptual independence; wording or method effects remain plausible. Contemporary conceptualizations distinguish positive and negative care-related experiences and recognize multidimensional structures within the broader literature on compassion satisfaction and compassion fatigue [4]. Nevertheless, whether the compassion and fatigue/exhaustion dimensions of the EFat-Com represent components of a single overarching construct remains an empirical question requiring further psychometric investigation. Replication with a new sample is needed.

In the primary model, more than 10 years of professional experience was associated with lower fatigue/exhaustion scores. This result is consistent with a study conducted in Palestine, which observed lower burnout in nurses with more than ten years of professional practice [21], and with another study that identified an inverse association between work experience and fatigue [23]. Along the same lines, another study reported greater secondary traumatic stress among professionals with less experience [7]. Although this relationship could reflect greater clinical familiarity and better coping resources, these mechanisms were not directly evaluated. In addition, the evidence is not uniform, as it has also been reported that there is no association between years of experience and burnout [1]. Therefore, experience should be considered a contextually associated factor, not a universal protective resource. In the expanded model, adjustment for age, resilience and SWLS substantially attenuated this estimate; experience should therefore not be presented as an association robust to all specifications.

The practice-environment estimate was negative, but its robust CI included zero in both primary and expanded fatigue/exhaustion models. The present data therefore does not provide a stable independent association, although organizational conditions remain relevant to interpretation. Previous studies have linked adequate staffing and resources, collaborative relationships, manager support, and positive work environments with higher compassion satisfaction and lower levels of burnout, emotional exhaustion, or secondary traumatic stress [12,21,22,39,40]. Likewise, systematic reviews have identified leadership, positive organizational culture, clinical supervision, and work environment as factors associated with lower burnout, although the certainty of the evidence remains moderate to low due to the predominance of cross-sectional designs and the heterogeneity of the studies [1,2]. Therefore, CF should not be interpreted only as an individual vulnerability, but as a phenomenon also related to emotional demands and available institutional resources.

Although resilience was not included in the primary regression models, exploratory sensitivity analyses showed that higher resilience was associated with higher compassion and lower fatigue/exhaustion. This finding is consistent with evidence identifying resilience as a relevant personal resource among care professionals. Higher resilience has been associated with greater prosocial behavior among professional caregivers [29]. From a broader perspective, a stronger sense of coherence has been associated with lower stress and adverse psychological outcomes and with greater well-being among care professionals [27]. Among nurses, higher emotional intelligence has been associated with greater compassion satisfaction and lower compassion fatigue [12], whereas greater perceived social support has been associated with lower compassion fatigue and burnout and greater compassion satisfaction [28]. Taken together, these findings support considering both personal and contextual resources when interpreting occupational outcomes among care professionals. However, because resilience was examined only in exploratory sensitivity analyses and was not included in the primary regression models, and given the cross-sectional design, this finding should be interpreted cautiously.

The limited explanatory capacity of the fatigue/exhaustion model (adjusted R^2^ = 0.080) indicates that the variables included in the primary model accounted for only a small proportion of the variability in this dimension. This finding suggests that other relevant factors not assessed in the present study may also contribute to fatigue/exhaustion. Evidence from nursing research on related, although conceptually dissimilar, occupational outcomes such as burnout has identified a broad range of individual, psychosocial, and organizational factors, including workload, staffing levels, work demands, social support, leadership, and working conditions [41,42]. Therefore, the observed associations should be interpreted cautiously and should not be considered a comprehensive explanation of fatigue/exhaustion.

Regarding the theoretical implications, the findings can be interpreted in a way that is consistent with the COR Theory, which conceives stress as a response to the threat or loss of valued resources and well-being as favored by their conservation and accumulation [30]. The presence of different association patterns for both dimensions supports the usefulness of analyzing them separately, although it does not demonstrate completely independent etiological mechanisms. The study also did not directly measure loss, gain, or investment of resources; therefore, the theory functions as an interpretative framework and not as an empirically contrasted model. Resilience was included in the expanded analyses as a personal resource and was associated with both dimensions, but its addition during revision and the cross-sectional design make these findings exploratory rather than a test of resource-change mechanisms.

In practical terms, the findings support the evaluation of multilevel strategies. At the individual level, interventions aimed at strengthening empathic communication skills and emotional regulation could be studied. At the organizational level, actions should be considered on leadership, autonomy, professional support, workloads, and availability of resources [1,2,12,24]. However, the cross-sectional design of the study does not allow us to establish that these interventions reduce CF. Their comparative effectiveness and sustainability require evaluation through longitudinal and intervention studies.

The results should be interpreted considering several limitations. First, the cross-sectional design prevents establishing causal relationships. Second, the use of self-report measures increases the risk of social desirability and common-method variance. Third, virtual recruitment through institutional, social and university networks could generate self-selection bias and limit the representativeness of the sample. The exclusion of professionals with a previous diagnosis of mental health problems could also introduce selection bias and potentially underestimate fatigue/exhaustion or other forms of occupational distress. Likewise, the use of convenience sampling, the predominantly female composition of the sample, and the concentration of participants in Lima limit the representativeness of the sample and the generalizability of the findings to the broader nursing population in Peru or other settings. Furthermore, empathy was assessed using a unidimensional subscale; therefore, the study could not distinguish the potentially different roles of cognitive, affective, or other specific components of empathy. In addition, the conceptual overlap between the empathy measure and the empathic-disposition content of the EFat-Com compassion dimension may have contributed to the magnitude of the observed association. Discriminant validity between these measures was not specifically examined in the present study. Complete-case regressions used 339 of 374 participants; unobserved differences between respondents with and without complete data may bias associations. Missingness mechanisms cannot be established from the available file. Small workplace and religious categories produce imprecise estimates, and the analytic sample included 19 respondents reporting no institutional employment, whose exclusion was examined in sensitivity analyses. EFat-Com respecification used the same sample and needs external replication; the PES-NWI five-factor solution was inadmissible. Model-based reliability therefore requires caution. Full and expanded models were adopted during revision, not prospectively preregistered. Stepwise selection showed optimism and instability in bootstrap analyses; this internal validation did not repeat the factor-model search. The primary fatigue/exhaustion model had adjusted R^2^ = 0.080, and its associations were sensitive to adjustment, indicating limited explanatory capacity and potential unmeasured factors.

## 5. Conclusions

Using the original EFat-Com subscales, empathy was consistently associated with higher compassion scores. Associations involving experience, job satisfaction, sex, and workplace characteristics were sensitive to model specification, and the fatigue/exhaustion model had limited explanatory capacity, suggesting that other relevant factors not assessed in this study may also contribute to variability in fatigue/exhaustion. Resilience was associated with both dimensions in exploratory expanded models. These findings support reporting the dimensions separately while recognizing measurement uncertainty, missing-data limitations, and the absence of causal inference. Given the convenience sampling strategy and the predominantly female, Lima-based sample, these findings should be considered context-specific and require replication in more representative and geographically diverse nursing populations. Independent replication and prospectively specified longitudinal studies are needed before translating these associations into intervention recommendations.

## Figures and Tables

**Table 1:** Frequencies and percentages of participants’ sociodemographic characteristics.

	*f*	%
*Sex*		
Male	44	11.76
Female	330	88.24
*Years of experience*		
Less than 5	84	22.46
Between 5 and 10 years	113	30.21
More than 10 years	177	47.33
*Religion*		
Catholic	284	75.94
Evangelical	48	12.83
Adventist	13	3.48
Agnostic	13	3.48
Other religion *	16	4.28
*Department of residence*		
Lima	277	74.06
Other department of Peru	97	25.94
*Work establishment*		
Hospital	227	62.88
Health center	59	16.34
Polyclinic	5	1.39
Clinic	32	8.86
Independent practice/Freelance	6	1.66
Other **	11	3.05
No institutional employment	21	5.82
*Job satisfaction*		
Satisfied	248	68.70
Neither satisfied nor dissatisfied	98	27.14
Dissatisfied	15	4.16

* Mormon, Jehovah’s Witness, and others ** Ambulance, physician’s office, and others. Percentages use available responses: *n* = 374 for sex, experience, religion and residence, and *n* = 361 for establishment and job satisfaction. The latter two variables each had 13 missing responses (3.48% of 374).

**Table 2:** Descriptive statistics and correlation matrix of nursing practice environment, empathy, and the dimensions of compassion fatigue.

Variable	M	SD	Min	Max	Spearman’s Rho		
					1	2	3
1. Nursing practice environment	2.70	0.57	1.10	4.00	-		
2. Empathy	4.76	0.82	2.40	6.00	0.23 **	-	
3. CF–Compassion	4.13	0.61	1.57	5.00	0.12 *	0.41 **	-
4. CF–Fatigue/exhaustion	2.78	0.69	1.17	5.00	−0.09	−0.1	−0.09

CF = Compassion fatigue. * *p* < 0.05, ** *p* < 0.001.

**Table 3:** Full Multiple Linear Regression Model for the Compassion Dimension of Compassion Fatigue.

Variable	b	LL	UL	β	*p*
Intercept	2.11	1.49	2.74	–	<0.001
Sex (Female)	0.20	−0.02	0.42	0.10	0.070
Experience (5–10 years)	0.04	−0.13	0.21	0.03	0.622
Experience (>10 years)	0.18	0.01	0.34	0.14	0.038
Establishment (Health center)	0.06	−0.09	0.21	0.04	0.459
Establishment (Polyclinic)	−0.52	−0.98	−0.07	−0.10	0.024
Establishment (Clinic)	0.05	−0.22	0.33	0.02	0.712
Establishment (Independent practice)	−0.53	−1.11	0.04	−0.11	0.068
Establishment (No institutional employment)	0.10	−0.14	0.35	0.04	0.409
Job satisfaction (Neutral)	−0.01	−0.16	0.14	0.00	0.940
Job satisfaction (Dissatisfied)	0.11	−0.27	0.49	0.04	0.562
Nursing practice environment	0.09	−0.04	0.22	0.08	0.190
Empathy	0.31	0.24	0.39	0.42	<0.001
Religion (Evangelical)	0.03	−0.13	0.19	0.01	0.742
Religion (Adventist)	−0.12	−0.66	0.41	−0.04	0.654
Religion (Agnostic)	0.07	−0.21	0.35	0.02	0.623
Religion (Other)	−0.01	−0.37	0.35	0.00	0.962

Note: *n* = 339; original 13-item scoring; adjusted R^2^ = 0.216. LL/UL = lower/upper limits of the HC3 95% CI; *p* uses HC3 inference. References: Male; <5 years; Hospital; Satisfied; Catholic. “Other” establishment had no complete outcome data. Polyclinic and independent practice estimates each use *n* = 5 and require caution.

**Table 4:** Full Multiple Linear Regression Model for the Fatigue/Exhaustion Dimension of Compassion Fatigue.

Variable	b	LL	UL	β	*p*
Intercept	3.14	2.52	3.77	–	<0.001
Sex (Female)	0.04	−0.25	0.33	0.02	0.783
Experience (5–10 years)	−0.02	−0.23	0.19	−0.01	0.833
Experience (>10 years)	−0.20	−0.40	−0.01	−0.15	0.043
Establishment (Health center)	0.22	−0.02	0.45	0.12	0.070
Establishment (Polyclinic)	0.50	−0.35	1.35	0.09	0.248
Establishment (Clinic)	0.04	−0.23	0.32	0.02	0.762
Establishment (Independent practice)	0.53	−0.18	1.24	0.09	0.142
Establishment (No institutional employment)	−0.14	−0.40	0.12	−0.05	0.294
Job satisfaction (Neutral)	0.23	0.06	0.39	0.15	0.009
Job satisfaction (Dissatisfied)	−0.02	−0.41	0.37	0.00	0.930
Nursing practice environment	−0.13	−0.29	0.03	−0.11	0.112
Empathy	−0.02	−0.10	0.07	−0.02	0.725
Religion (Evangelical)	0.07	−0.15	0.29	0.03	0.528
Religion (Adventist)	−0.20	−0.67	0.26	−0.06	0.395
Religion (Agnostic)	−0.14	−0.68	0.39	−0.04	0.598
Religion (Other)	0.32	−0.04	0.68	0.09	0.084

Note: *n* = 339; original 13-item scoring; adjusted R^2^ = 0.080. LL/UL = lower/upper limits of the HC3 95% CI; *p* uses HC3 inference. References: Male; <5 years; Hospital; Satisfied; Catholic. “Other” establishment had no complete outcome data. Polyclinic and independent practice estimates each use *n* = 5 and require caution.

## Data Availability

Data are available upon request addressed to the corresponding author due to privacy restrictions.
